# NMR and ESIMS data for bisabolane-type sesquiterpenoids

**DOI:** 10.1016/j.dib.2019.104780

**Published:** 2019-11-11

**Authors:** Shinji Ohta, Yasunori Yuasa, Nobuwa Aoki, Emi Ohta, Tatsuo Nehira, Hisashi Ômura, Mylene M. Uy

**Affiliations:** a Graduate School of Integrated Sciences for Life, Hiroshima University, 1-7-1 Kagamiyama, Higashi-Hiroshima, 739-8521, Japan; b Nagahama Institute of Bio-Science and Technology, 1266 Tamura-cho, Nagahama, Shiga, 526-0829, Japan; c Department of Chemistry, Mindanao State University-Iligan Institute of Technology, Iligan City, 9200, Philippines

**Keywords:** *Angelica keiskei*, Seed, Bisabolane sesquiterpenoid, NMR, ESIMS

## Abstract

The data presented here are related to the research paper entitled “Norbisabolane and bisabolane sesquiterpenoids from the seeds of *Angelica keiskei*” [1]. In this data article, we provide 1D and 2D nuclear magnetic resonance (NMR) spectroscopy and electrospray ionization mass spectrometry (ESIMS) data of three undescribed norbisabolane- and bisabolene-type sesquiterpenoids, ashitabaol B-D isolated from the seeds of *Angelica keiskei*.

**Table:** Specifications Table

Subject area	*chemistry*
More specific subject area	*natural products*
Type of data	*Figure*
How data was acquired	*NMR spectroscopy: JEOL A400; ESIMS: Shimadzu LCMS-IT-TOF and Thermo Fisher Scientific LTQ Orbitrap XL.*
Data format	*Raw and analyzed*
Experimental factors	*The undescribed sesquiterpenoids were purified by column chromatography.*
Experimental features	*The isolated compounds were characterized by ESIMS and NMR spectroscopy*
Data source location	*Higashi-Hiroshima, Japan*
Data accessibility	*Data are available with this article*
Related research article	S. Ohta, Y. Yuasa, N. Aoki, E. Ohta, T. Nehira, H. Ômura, M. M. Uy, Norbisabolane and bisabolane sesquiterpenoids from the seeds of *Angelica keiskei, Phytochemistry Letters 33 (2019) 94–101.*

**Table:** 

**Value of the Data** • The data presents NMR data and ESIMS data of newly isolated sesquiterpenoids and could be used by other researchers. • The provided information on the spectroscopic data of sesquiterpenoids could be useful for the analysis of spectra and determination of the structure of other sesquiterpenoids. • This data can serve as a benchmark for other researchers to elucidate the structures of sesquiterpenoids

## 1 Data

**Fig. 1:**
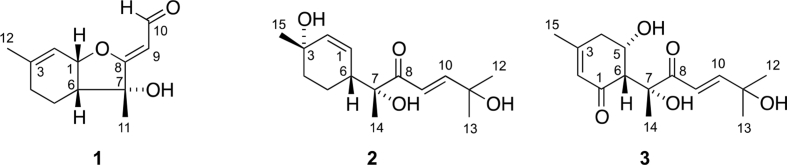
Structures of norbisabolane- and bisabolane-type sesquiterpenoids isolated from the seeds of *A. keiskei*.

**Fig. 2:**
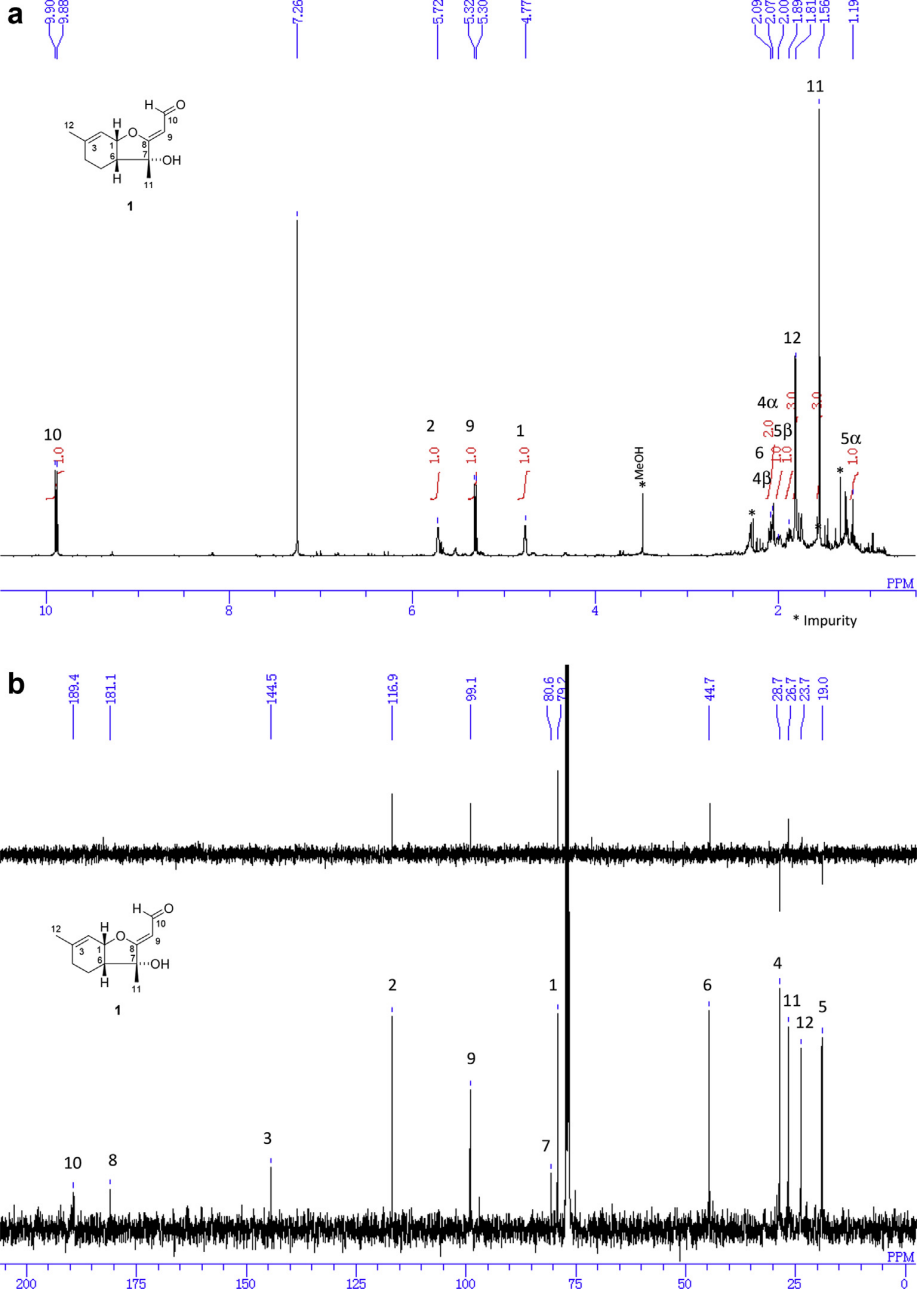

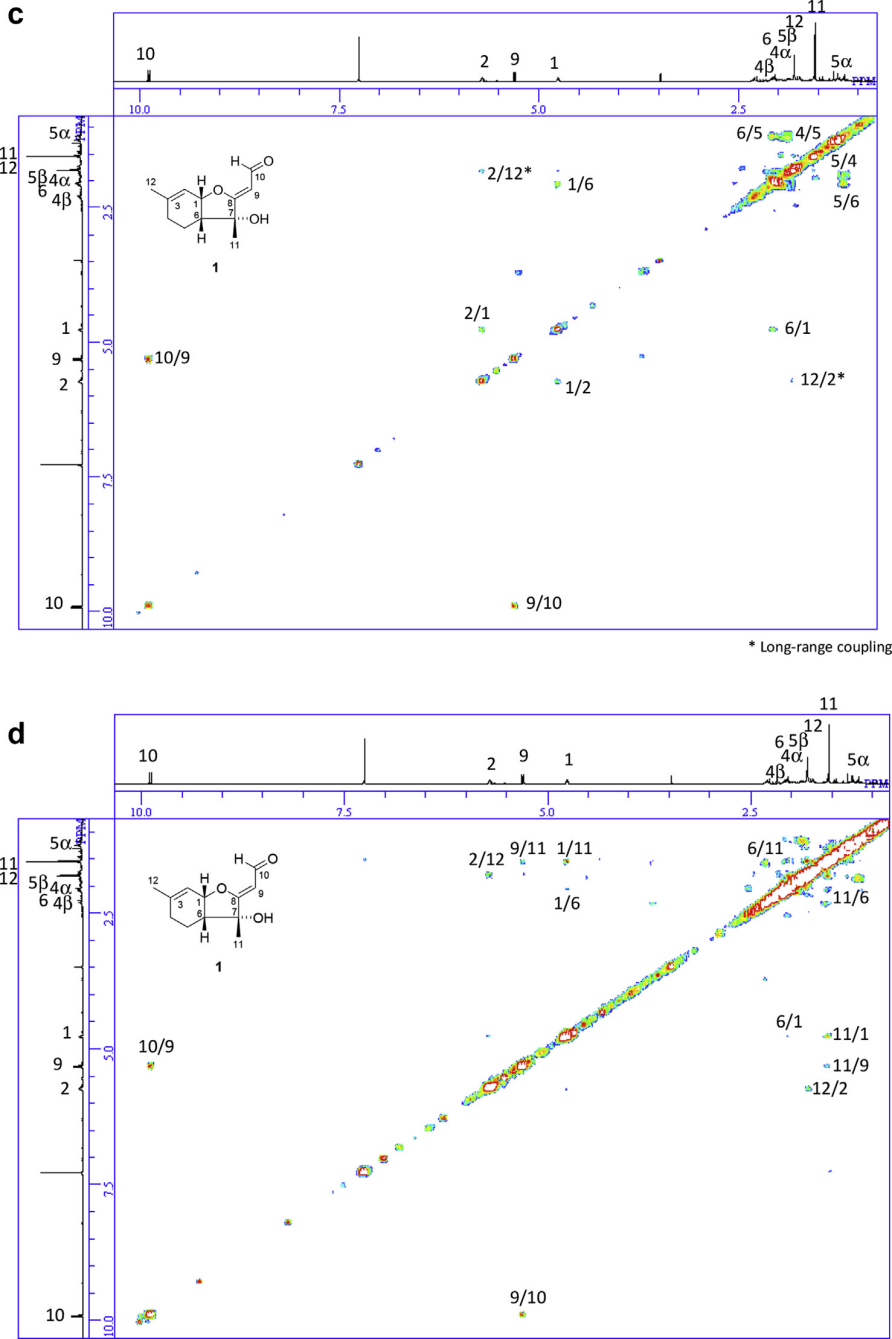

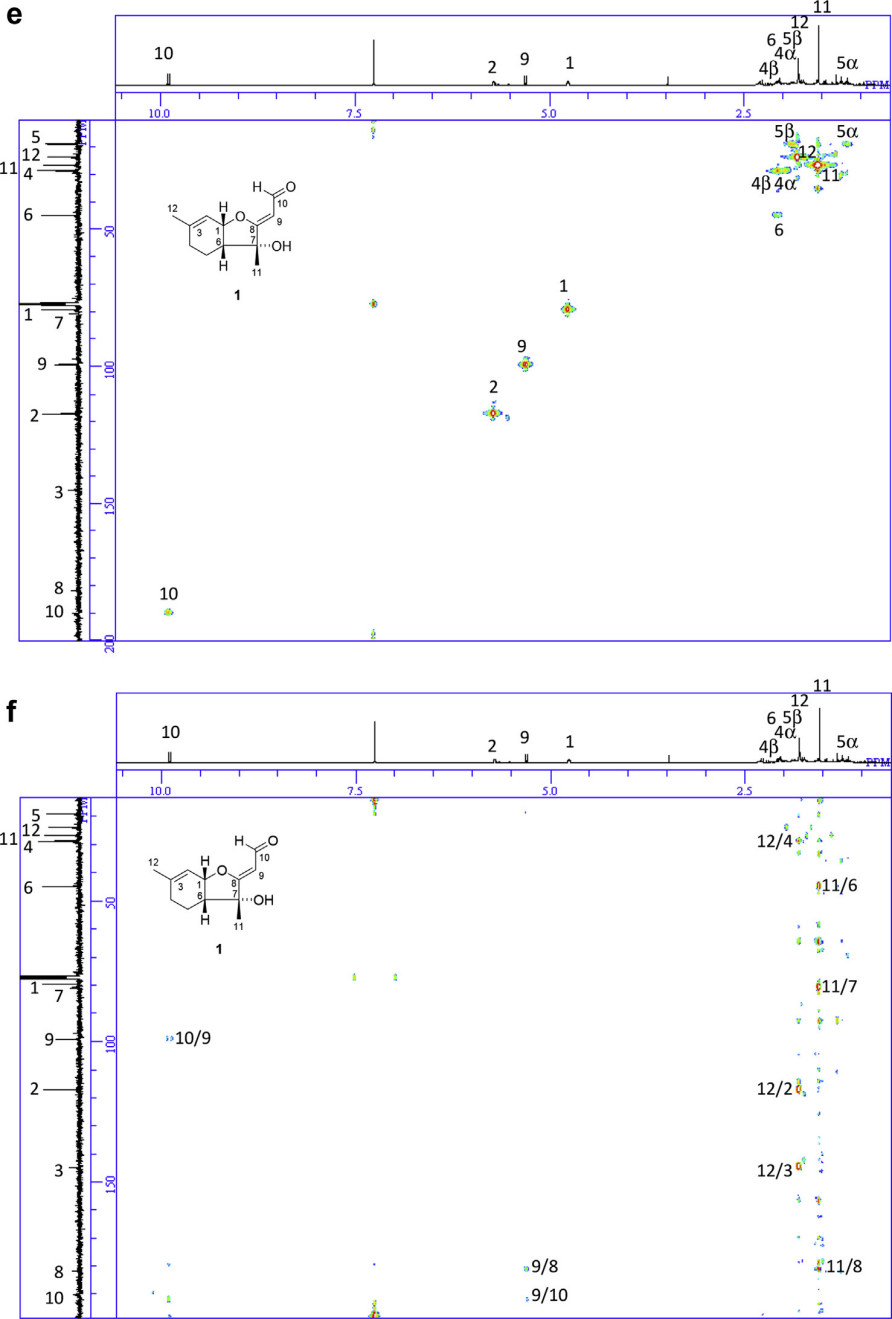

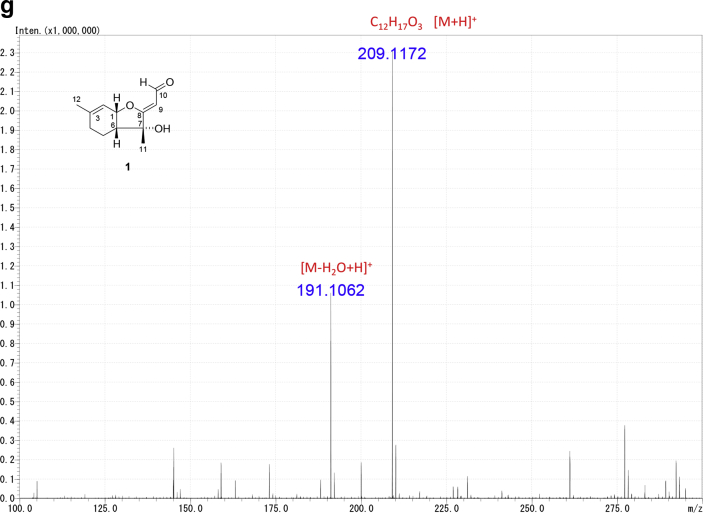
**a**. ^1^H NMR (400 MHz, CDCl_3_) of **1**, **b**. ^13^C NMR and DEPT (100 MHz, CDCl_3_) of **1**, **c**. ^1^H–^1^H COSY of **1**, **d**. NOESY of **1**, **e**. HSQC of **1**, **f**. HMBC of **1**, **g**. (+)ESIMS of **1**.

**Fig. 3:**
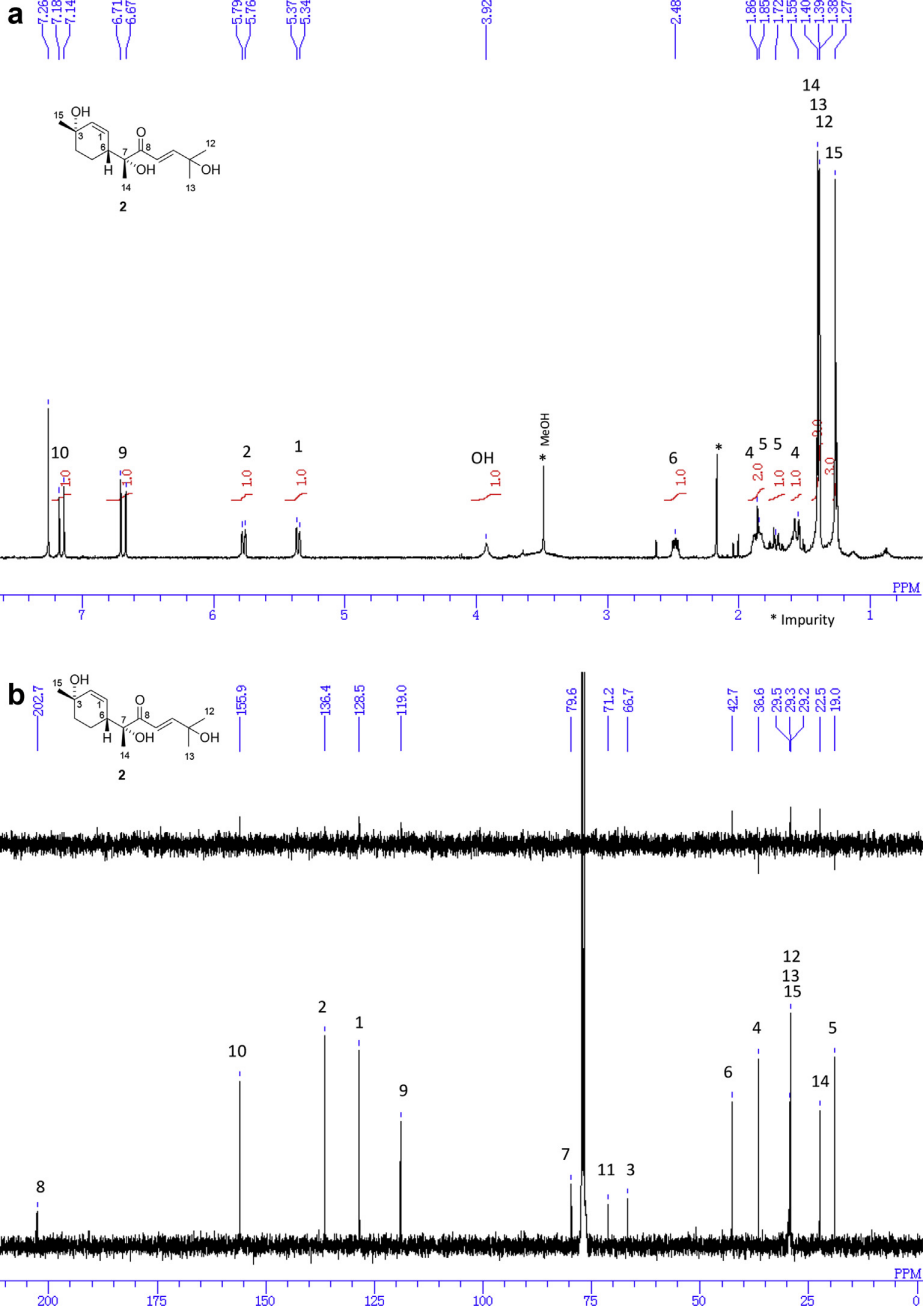

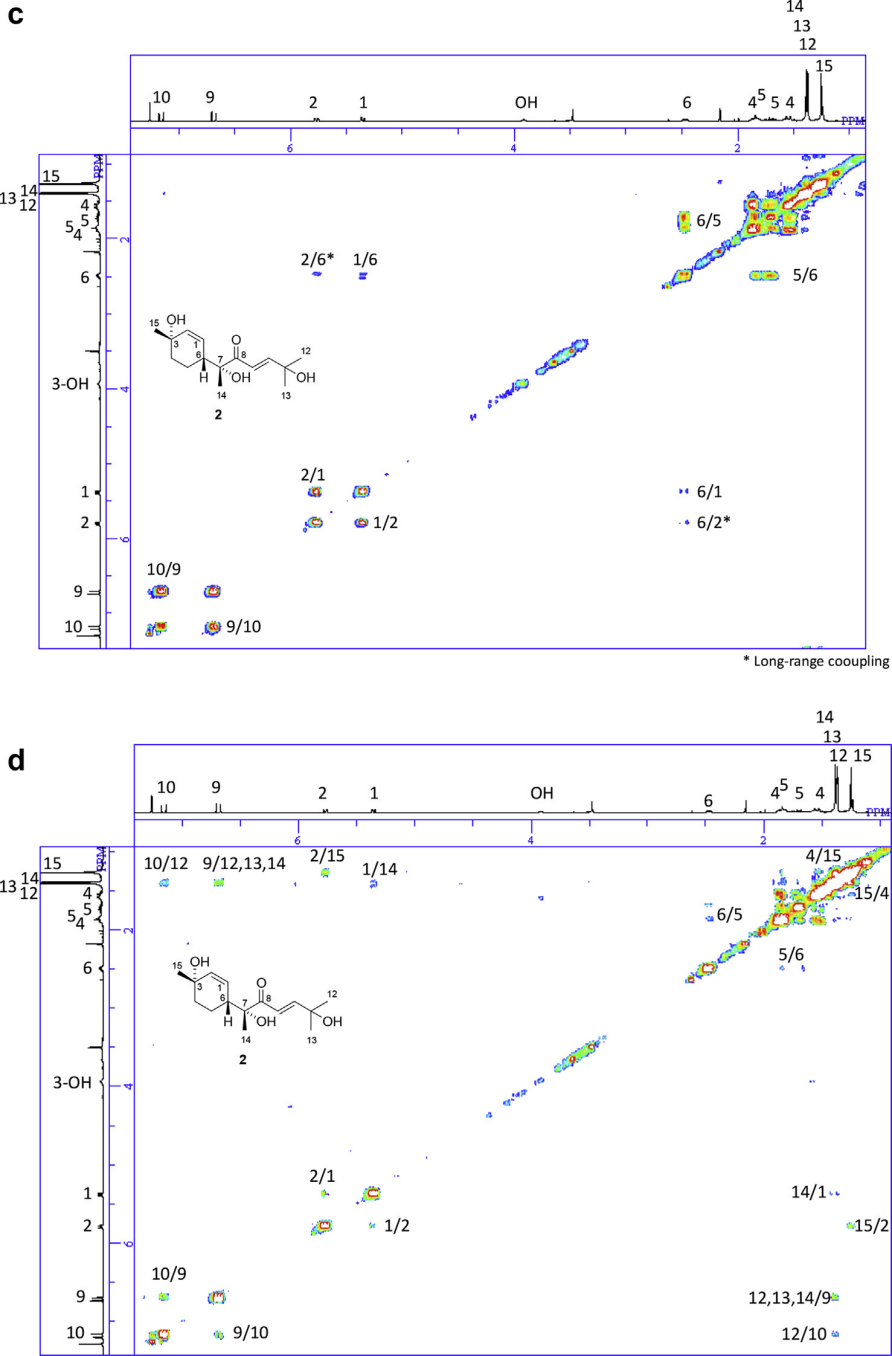

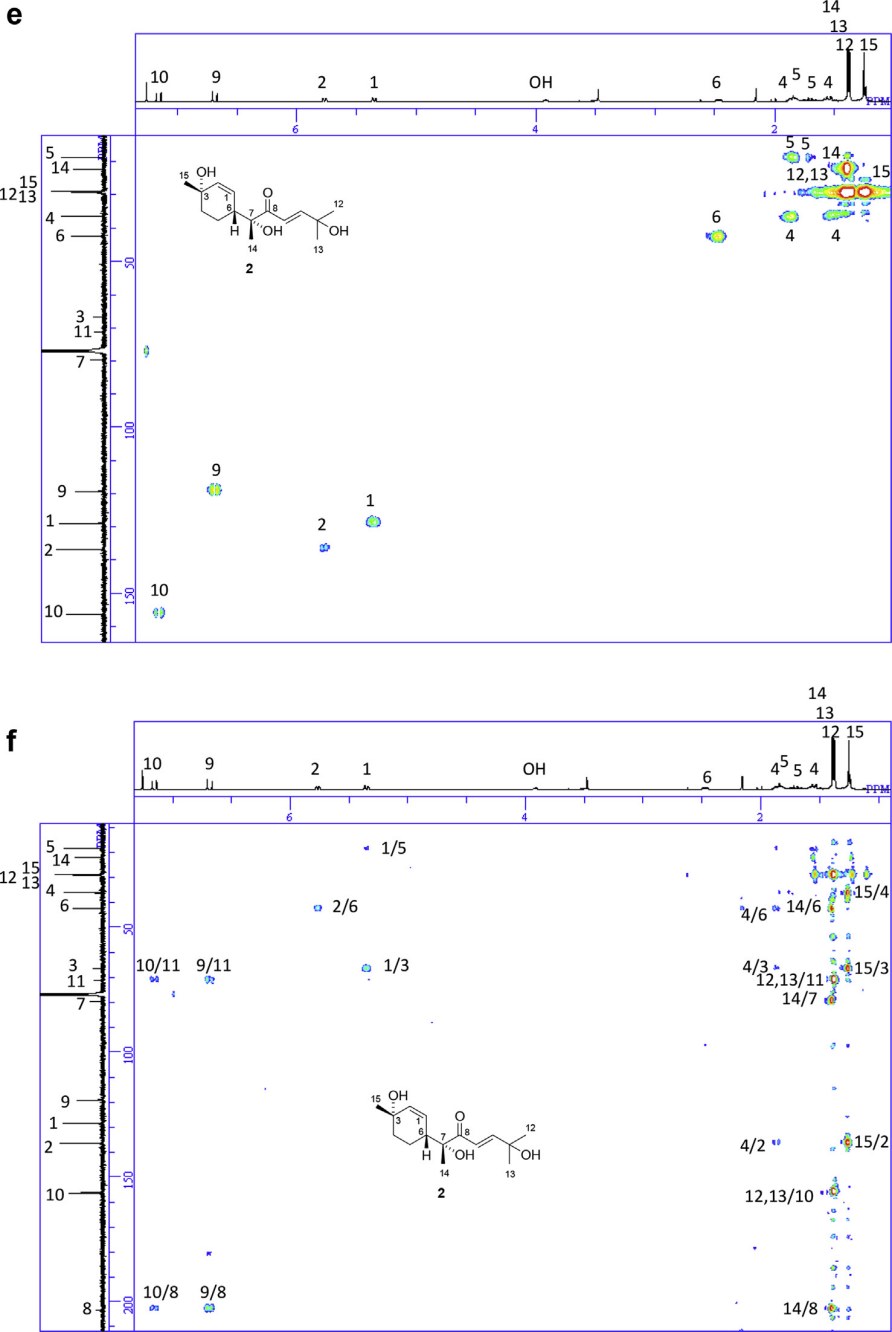

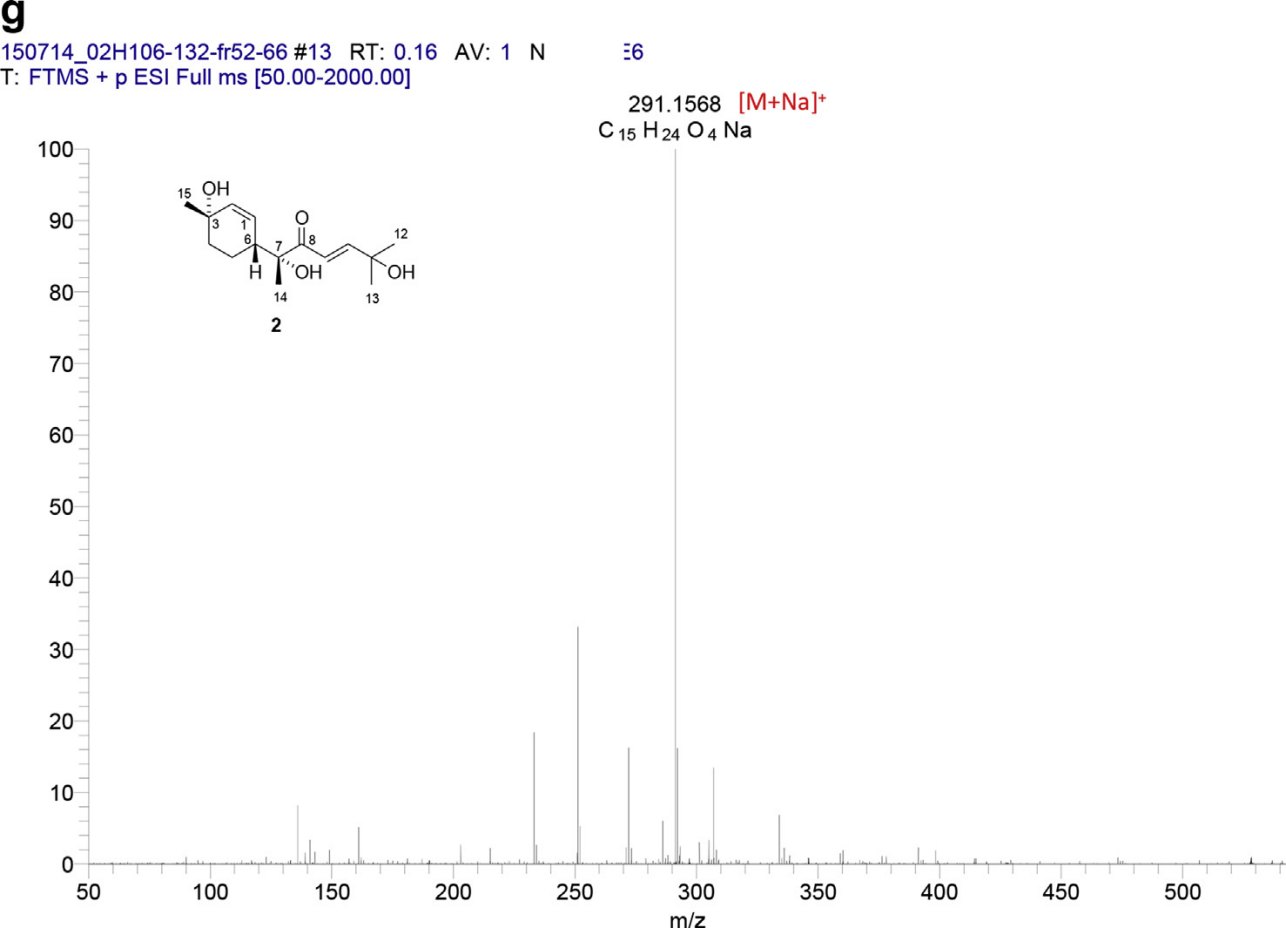
**a**. ^1^H NMR (400 MHz, CDCl_3_) of **2**, **b**. ^13^C NMR and DEPT (100 MHz, CDCl_3_) of **2**, **c**. ^1^H–^1^H COSY of **2**, **d**. NOESY of **2**, **e**. HSQC of **2**, **f**. HMBC of **2**, **g**. (+)ESIMS of **2**.

**Fig. 4:**
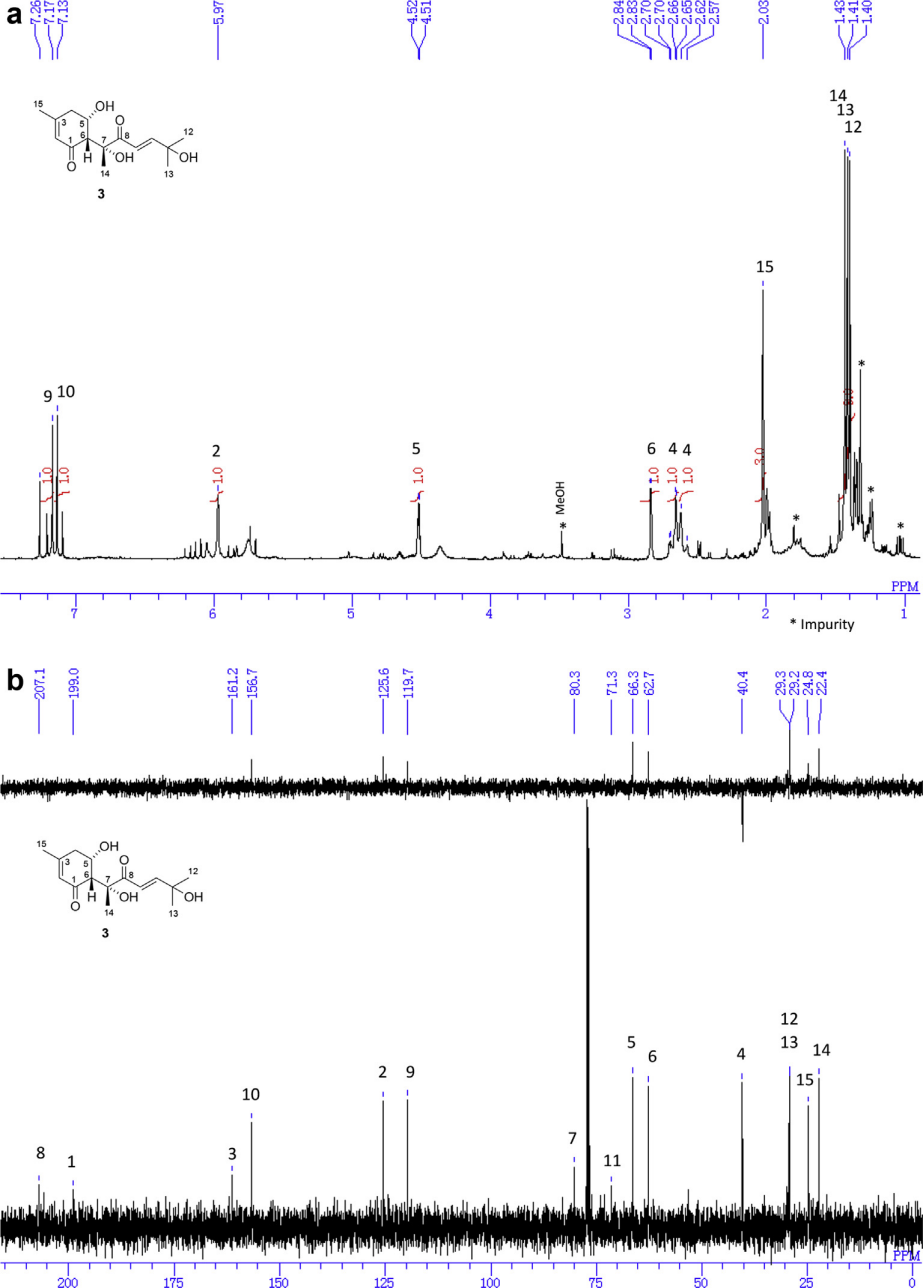

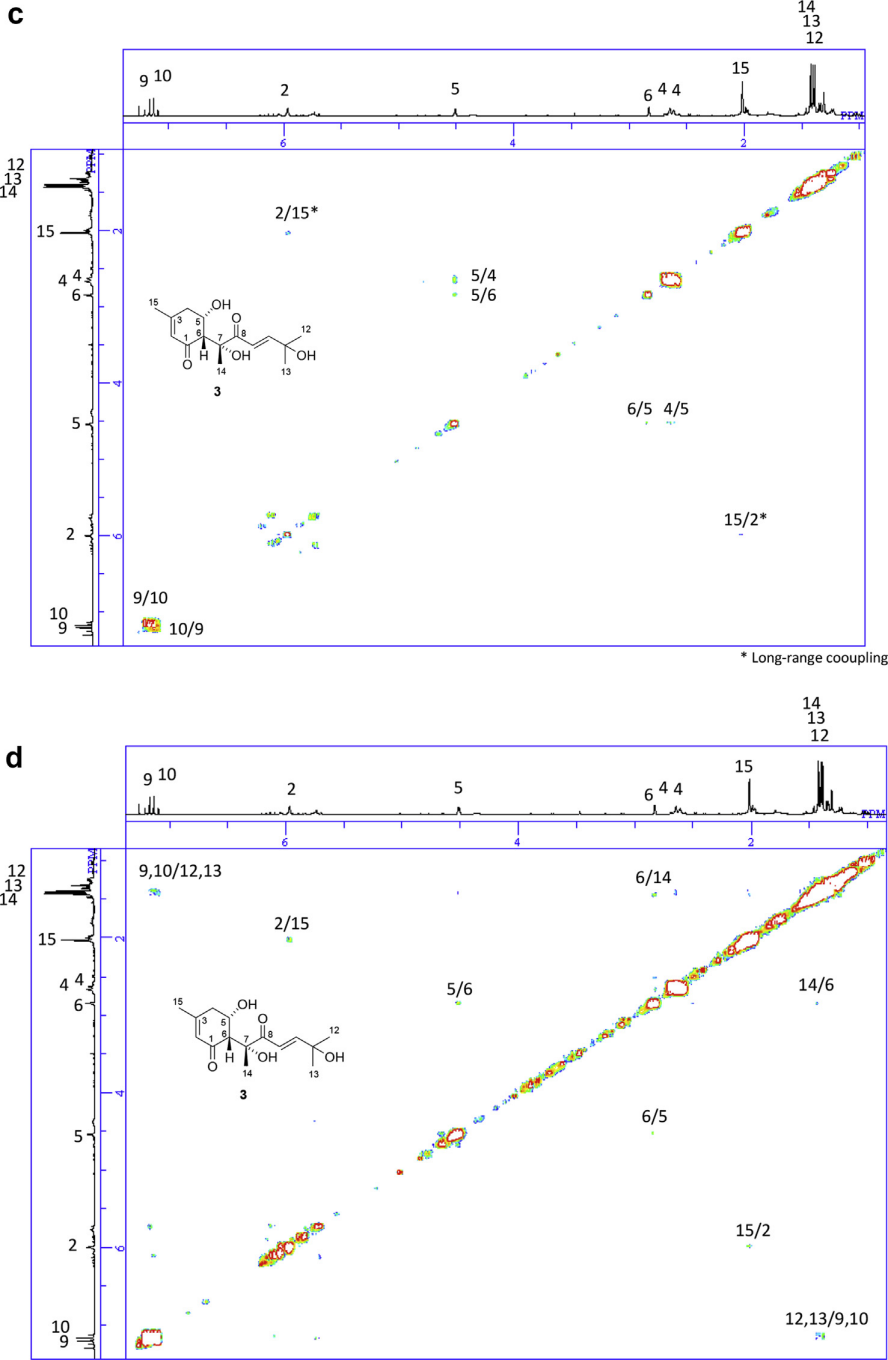

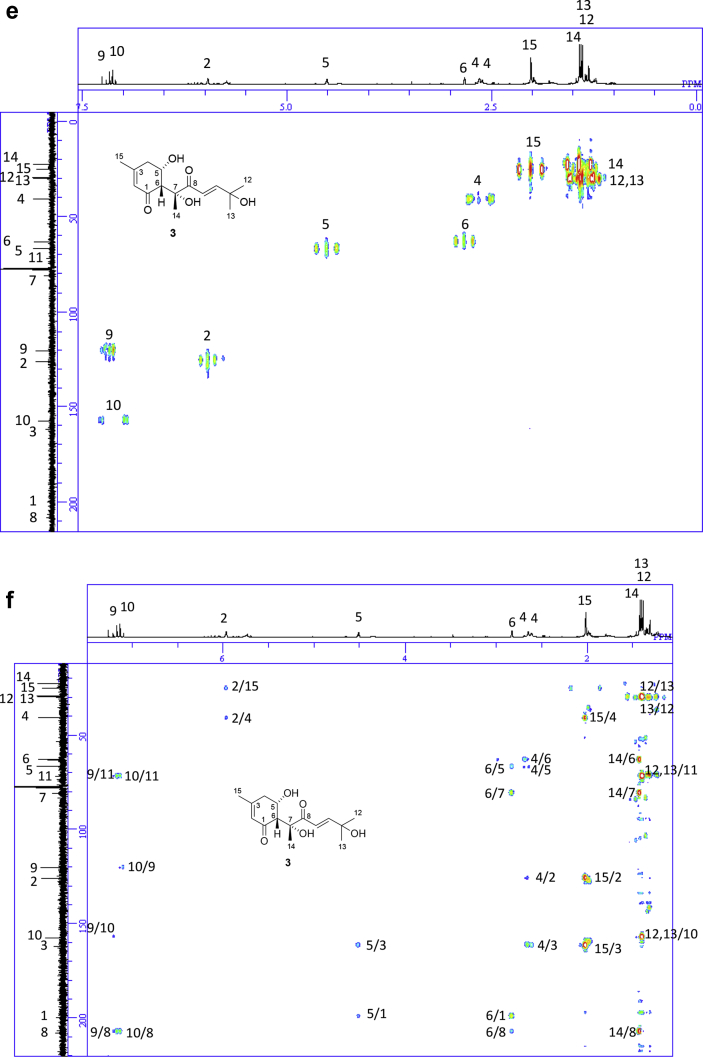

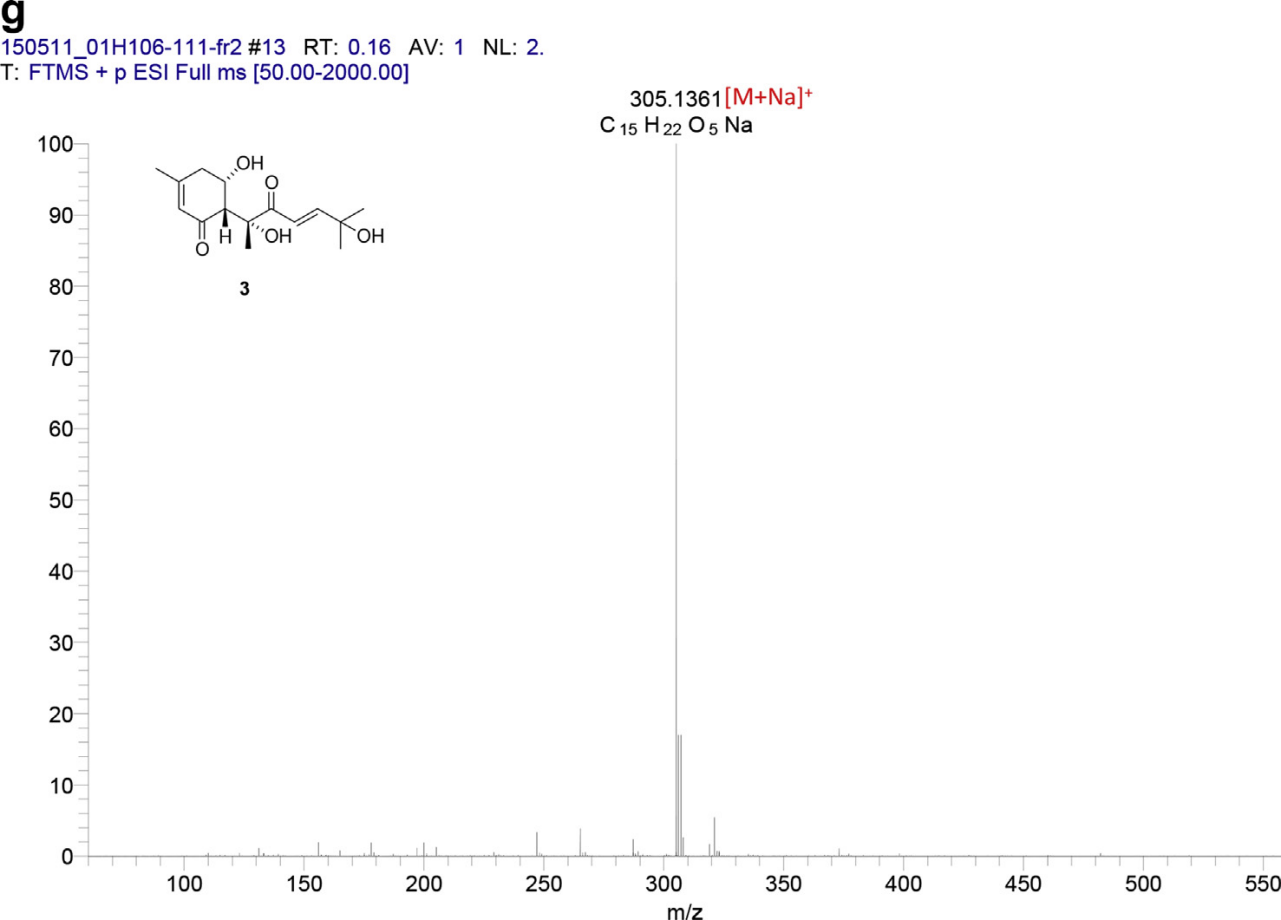
**a**. ^1^H NMR (400 MHz, CDCl_3_) of **3**, **b**. ^13^C NMR and DEPT (100 MHz, CDCl_3_) of **3**, **c**. ^1^H–^1^H COSY of **3**, **d**. NOESY of **3**, **e**. HSQC of **3**, **f**. HMBC of **3**, **g**. (+)ESIMS of **3**.

The data set presented in this article focuses on characterization of the norbisabolane- and bisabolane-type sesquiterpenoids described in Ref. [1]. The article provides the information on the spectroscopic data of the sesquiterpenoids **1**–**3** isolated from the seeds of *Angelica keiskei* (Fig. 1). The ^1^H NMR spectra of **1**–**3** are shown in Fig. 2, Fig. 3, Fig. 4a, respectively. The ^13^C NMR and DEPT spectra of **1**–**3** are shown in Fig. 2, Fig. 3, Fig. 4b, respectively. 2D ^1^H–^1^H COSY spectra of **1**–**3** are shown in Fig. 2, Fig. 3, Fig. 4c, respectively. 2D NOESY spectra of **1**–**3** are shown in Fig. 2, Fig. 3, Fig. 4d, respectively. 2D ^1^H–^13^C heteronuclear single quantum coherence (HSQC) spectra of **1**–**3** are shown in Fig. 2, Fig. 3, Fig. 4e, respectively. 2D ^1^H–^13^C heteronuclear multiple-bond correlation (HMBC) spectra of **1**–**3** are shown in Fig. 2, Fig. 3, Fig. 4f, respectively. ESIMS data of **1**–**3** are shown in Fig. 2, Fig. 3, Fig. 4g, respectively. Analyses of the spectra of **1**–**3** are described in the research article [1].

## 2 Experimental design, materials, and methods

NMR spectra were acquired using a JEOL A400 spectrometer (400 MHz for ^1^H, 100 MHz for ^13^C). ^1^H and ^13^C NMR chemical shifts were referenced to residual solvent peaks: *δ*_H_ 7.26 (residual CHCl_3_) and *δ*_C_ 77.0 for CDCl_3_. ESIMS were carried out using a Shimadzu LCMS-IT-TOF mass spectrometer and a Thermo Fisher Scientific LTQ Orbitrap XL mass spectrometer at the Natural Science Center for Basic Research and Development (N-BARD), Hiroshima University.

## 3 Sesquiterpenoids **1–3**

### 3.1 (Z)-2-((3R,3aR,7aS)-3-Hydroxy-3,6-dimethyl-3,3a,4,5-tetrahydrobenzofuran-2(7aH)-ylidene)acetaldehyde (ashitabaol B) (**1**)

1D NMR, 2D NMR, and ESIMS spectra of the compound **1** are shown in Fig. 2a–g.

### 3.2 (E)-2,6-Dihydroxy-2-(4-hydroxy-4-methylcyclohex-2-en-1-yl)-6-methylhept-4-en-3-one (ashitabaol C) (**2**)

1D NMR, 2D NMR, and ESIMS spectra of the compound **2** are shown in Fig. 3a–g.

### 3.3 (5S,6R)-6-((R,E)-2,6-Dihydroxy-6-methyl-3-oxohept-4-en-2-yl)-5-hydroxy-3-methylcyclohex-2-enone (ashitabaol D) (**3**)

1D NMR, 2D NMR, and ESIMS spectra of the compound **3** are shown in Fig. 4a–g.
